# St. Mary's Hospital, Paddington

**Published:** 1892-12-24

**Authors:** 


### St. Mary's Hospital, Paddington.

### THE CLARENCE MEMORIAL WING.

Their Royal Highnesses the Prince of Wales and the Princess Maud on their arrival at St. Mary's Hospital on Saturday last were received by the Duke of York, President of the Hospital, the Duke of Cambridge, and the Reception Committee. The Duke of York, in a short speech, which was very well delivered, thanked his Royal father in the name of the Governors for his presence on the occasion, and after Mr. Field, Chairman of the Extension Committee, had read an address, the Prince of Wales said: "I must thank my son, as President of St. Mary's Hospital, for the kind welcome he has extended to me on this occasion, and Mr. Field in the name of the Governors of this great hospital. This, my first public act since the great sorrow which fell on our family, is a satisfaction to me, insomuch as the foundation stone I am about to lay to-day will bear the name of my ever-to-be-lamented son. With you I also deeply deplore that the Princess of Wales is unable to accompany me to-day; but I feel convinced that you will all understand and appreciate her absence on this occasion. I am glad to think that my youngest daughter is with me and will take the purses which will be given to-day. I may say that the members of my family have been now for some considerable time associated with this hospital, the idea first emanating in the very year of my birth, 1841, when it was considered necessary, on account of the large district about Paddington and North Kensington, to build a hospital. Six years after, my father laid the first stone, twenty years later, I remember well, I laid a stone to commemorate the enlargement of the hospital, and still more recently my sister, Princess Louise, laid the stone of the Mary Stamford Wing. Like all hospitals in this country much money is here needed, and it is not always easy to get it. I know also that your original scheme was to have 380 beds, and I regret that you have only 281. I sincerely trust that in this new wing you will be able to have all that is requisite. . . . There is much more I might say, but I will only add that I know what a satisfaction it has been to my son to accept the invitation to be President of this great hospital, and to associate himself permanently with it, not only on account of its great philanthropic objects, but because he was anxious to pay some debt of gratitude to the distinguished physician who is so intimately connected with this hospital, Dr. Broadbent, for the great care with which he attended him during his long and serious illness last year. I thank you, ladies and gentlemen, for having so kindly listened to the remarks I have made. I assure you again that it is a satisfaction to me to lay the foundation-stone of the new wing." The Duke of York handed a silver trowel to his father, requesting him to lay the stone, which was immediately done. The inscription read as follows: "This stone was laid by H.R.H. Albert Edward, Prince of Wales, on 17th December, 1892, in memory of H.R.H. Albert Victor Christian Edward, Duke of Clarence and Avondale, who died 14th January, 1892." The Bishop of Marlborough offered up prayer. Mr. Field, Dean of the Medical School, then presented eighteen students to the Prince of Wales, who handed to each the certificate awarded to him. Princess Maud received purses from ladies and children, containing contributions to the Clarence Memorial Fund. This was one of the prettiest features of the day's proceedings. Many of the children were such charming little creatures, mere babies, indeed, but they offered their dainty purses with a graceful simplicity whilst exhibiting a certain conscious pride as the sums contained in their bags were announced by the Secretary, Mr. Ryan. The Prince of Wales laid down a cheque for £50 as his own contribution, and the Duke of York had already subscribed £25 towards the fund. The sums contained in the purses amounted to over £1,100. Much enthusiasm was exhibited both within and without the tent, and it was evident that the first public re-appearance of the Prince of Wales and the members of his family was cordially appreciated. The Duke of Cambridge, whose unwearied interest in the welfare of all hospitals is widely known, was also warmly cheered on leaving the building.

